# Construction, Expression and Evaluation of Recombinant VP2 Protein for serotype-independent Detection of FMDV Seropositive Animals in Egypt

**DOI:** 10.1038/s41598-019-46596-9

**Published:** 2019-07-12

**Authors:** Reda Salem, Alaa A. El-Kholy, Omar A. Omar, Mohamed N. Abu el-naga, Mohamed Ibrahim, Gamal Osman

**Affiliations:** 1 Agricultural Genetic Engineering Research Institute (AGERI), ARC, 12619 Giza, Egypt; 2 Veterinary Sera and Vaccines Research Institute (VSVRI), ARC, Abbassia, Cairo, Egypt; 3 Radiation microbiology department, National Center for Radiation Research and Technology, Atomic Energy Authority, 11787, Cairo, Egypt; 4 Department of Molecular and Cell Biology, University of Texas at Dallas, Richardson, TX 75083 USA; 5 Biology Department, Faculty of Science, Umm-Al-Qura University, Mecca, 673 Saudi Arabia; 6 Research Laboratories Center, Faculty of Applied Science, Umm Al-Qura University, Mecca, Saudi Arabia

**Keywords:** Environmental biotechnology, Expression systems

## Abstract

Foot-and-mouth disease virus (FMDV) is one of the most devastating viral pathogens of cloven-hoofed animals. The detection of antibodies (Ab) against FMDV structural proteins (SP) using virus neutralization test (VNT) and liquid-phase blocking ELISA (LPBE) is the standard procedure in use for monitoring seroconversion in animals post vaccination, the prevalence of infection-surveillance, proving clinical cases and seronegative status of FMDV-free/naïve-animals prior transportation. However, due to variations within SP of FMDV serotypes, each serotype-specific Ab should be detected separately which is laborious and time-consuming. Accordingly, it is crucial to develop a sensitive, rapid, and accurate test capable of detecting FMDV-specific Ab, regardless its serotype. This study describes the heterologous expression of VP2 protein in *E*. *coli*, and its evaluation as a capture antigen in a simple indirect ELISA for serotype-independent detection of anti-FMDV Ab. Sequence analysis revealed that the VP2-coding sequence is considerably conserved among FMDV serotypes. The recombinant VP2 (rVP2), a 22 kDa polypeptide, was purified to near homogeneity by affinity chromatography under native conditions. Immunoreactivity of the rVP2 was confirmed by using a panel of positive sera including sera from animals vaccinated with the local trivalent vaccine and guinea pig FMDV antiserum, which is routinely used as tracing/detecting Ab in LPBE testing. The results obtained from the VP2-based ELISA were comparable to those determined by VNT and LPBE standard diagnostic assays. Specificity and sensitivity of rVP2 in capturing anti-FMDV Ab were 98.3% and 100%, respectively. The developed VP2-ELISA is proved reliable and time-efficient assay for detection of FMDV seropositive animals, regardless the FMDV serotype that can be implemented in a combination with VNT and/or LPBE for rapid diagnosis of an ongoing FMDV infection.

## Introduction

Foot-and-mouth disease (FMD) is a severe ailment affecting livestock. There are seven antigenically and genetically divergent serotypes of the etiological agent FMD virus (FMDV): O, A, C, Asia 1, and the Southern African Territories SAT 1, SAT 2, and SAT 3^1^. FMDV particles are made up of an icosahedral capsid each consisting of sixteen repeats of four structural proteins (SPs) VP1, VP2, VP3, and VP4, encapsulating a single-stranded, positive-sense RNA genome^2^. In addition to the structural proteins, during viral infection, a number of non-structural viral proteins (NSP) are also made in the infected cells^3^.

FMD has been endemic in Egypt since the 1970s with three prevalent serotypes: A, O, and SAT 2^4–6^. Serotype A was responsible for a critical outbreak of FMD in 2006 and serotype SAT2 caused an extensive outbreak in 2012. In our region, the possible introduction of new serotypes and sub-serotypes of FMDV by uninterrupted movement of animals across borders is a major persistent threat with substantial diverse endemic types of FMDV. Accordingly, vaccines containing these serotypes have been in use to immunize nearly all cloven-hoofed animals in Egypt. In Endemic areas, like Egypt, appropriate FMD inactivated vaccines play a key role in the control programs of FMD^7^.

 Detection of FMDV can only be confirmed through a serological examination. The serological tests for FMDV are performed for any of these purposes: confirm suspected cases, certify individual animals prior to movement for trade, substantiate absence of infection, and demonstrate the efficacy of vaccination. The serological tests for FMDV are of two types: one detects antibodies to NSP and the other detects antibodies to SP. The SP based tests detect antibodies elicited by infection or vaccination, as in the liquid-phase blocking ELISA (LPBE)^8,9^ and the virus neutralization test (VNT)^10^. The SP based tests are appropriate for monitoring immunity conferred by vaccination in the field, confirming previous or ongoing infection in non-vaccinated animals, to certify animals prior to transportation (for trade purposes). However, both techniques (LPBE and VNT) use inactivated live virus that poses certain health risks and they are also time-consuming, technically challenging, and have a significant rate of false positive reactions. Therefore, novel tests that harness the recombinant DNA technology to express target proteins in eukaryotic or prokaryotic systems are under development^11,12^.

The FMDV structural proteins are very diverse and any change in their composition enables FMDV to escape the host immune system^13^, which complicates the detection of antibodies against each serotype. Therefore, it is crucial to have a sensitive, rapid and reproducible laboratory test capable of detecting the FMDV antibodies regardless of the serotypes.

VP2 protein is a relatively conserved region among the FMDV serotypes; however, some substitutions in its amino acids have resulted in a significant antigenic diversity, indicating that VP2 harbors essential antigenic epitopes^14^.

Therefore, the key objectives of this endeavor are to construct and express a recombinant VP2 protein to be utilized as a coating antigen in a simple local indirect ELISA for detection of antibodies against FMDV regardless of its serotype, as an alternative to inactivated FMD viral antigen for confirming previous or ongoing infection in non-vaccinated animals, to certify animals prior to movement (trade purposes), and sero-monitoring of FMDV vaccinated animals that might be functional for assessment of FMD vaccination programs.

## Results

### Preparation of VP2 conserved antigen

#### Selection and cloning

**Figure 1:**
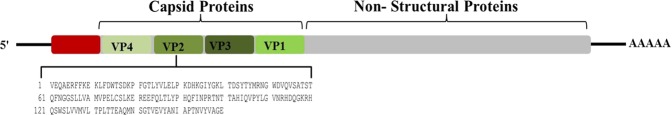
Schematic diagram showing the arrangement of the FMDV genome as well as the VP2 amino acids sequence. The 5′ half of FMDV genome codes for the structural proteins VP4, VP2, VP3, and VP1, while the 3′ half codes for the non-structural proteins such as protease and RNA-dependent RNA polymerase (RdRP–1) coding genes. The deduced amino acid sequence of VP2 capsid protein is shown in grey color.

**Figure 2:**
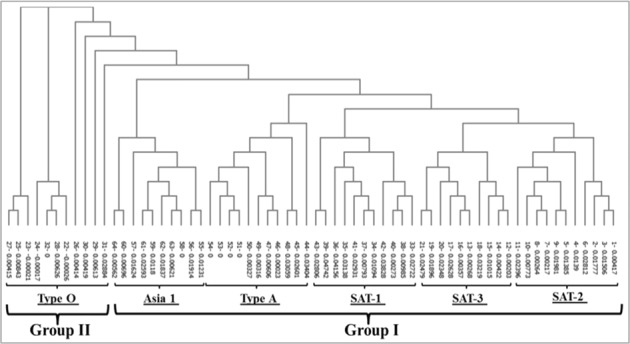
Bootstrapped neighbor-joining tree of 64 FMDV isolates. The tree was constructed based on the alignment of the VP2 amino acids sequence. Multiple sequence alignments were performed using the Clustal W algorithm. The retrieved sequences are assigned numbers 1 to 64 and the bootstrap values are presented beside each sequence ID. The VP2 capsid protein obtained from a local SAT 2 isolate (gb|AAZ83686) was used as a template. The amino acid sequence of the VP2 capsid protein from several isolates belonging to SAT 1, SAT 2, SAT 3, type A, and type O were retrieved from the GenBank (NCBI) and used for all alignments.

**Figure 3:**
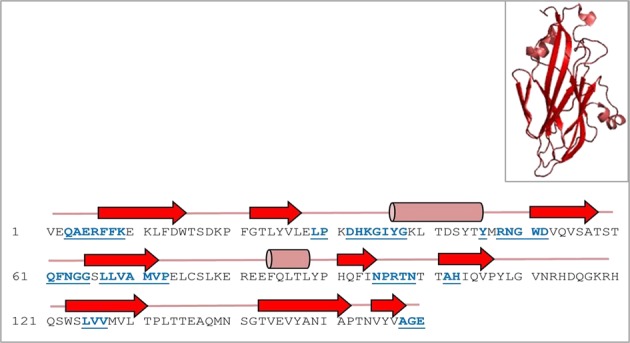
Secondary structure prediction and three-dimensional (3D) model of the VP2 capsid protein. The Jpred 4 database was used to predict the secondary structure (residues, 1–160) obtained from a local SAT 2 isolate (gb|AAZ83686). Red arrows highlight predicted β-strands. Cylinders and lines, shown in light purple color, highlight the α-helices and connecting loops, respectively. Amino acid residues in blue and underlined represent highly conserved sequence among different FMDV types. The 3D structure for the VP2-capsid protein, in ribbon format, is shown as an insert in the upper right corner of the figure.

The amino acid sequence of the FMDV VP2 capsid protein of the Egyptian SAT 2 isolate, (gb│AAZ83686) (Fig. 1) was used as the template to determine the homogeneity between several FMDV isolates representing the seven prevalent FMDV serotypes. Based on the results, the FMDV serotypes were clustered into– Group I serotypes including SAT 1, SAT 2, SAT 3, Asia 1, and Type A, and Group II that included Type O. The relatedness of these serotypes is summarized in the bootstrapped neighbor-joining tree of 64 FMDV isolates as shown in Fig. 2. Type C FMDV was not included in this study, since it has a consensus sequence with VP2, one can speculate the same relatedness. SAT 1, 2, 3, and type A were most similar, type O was divergent, and type Asia 1 was more closely related to SAT 1, 2, 3, and type A than to type O. The data revealed VP2-coding sequence to be highly conserved among the four FMDV capsid proteins. Furthermore, the prediction of secondary and three-dimensional (3D) structures of VP2 showed that certain motifs were highly conserved among six different FMDV types. These motifs mainly form loops connecting the β-strands and α-helices in the VP2-capsid protein (Fig. 3). Based on these findings, the VP2-coding sequence was modified and optimized to match the codon usage preference of *E*. *coli*. This modified sequence was synthesized and cloned into the P^Gex–4t1^ vector.

### Expression of recombinant VP2-capsid protein

**Figure 4:**
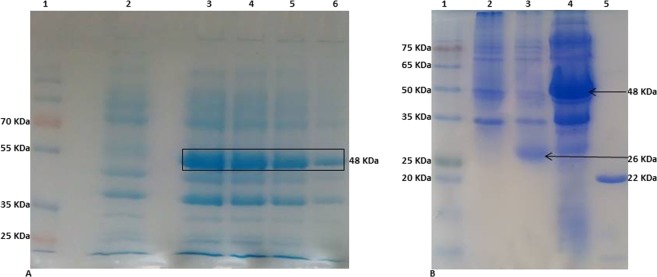
(**A**) SDS-page analysis visualizing the expressed VP2-protein fused to GST-protein (VP2 + GST) during expression, 1: protein marker, 2: cell lysate from non-transformed *E*. *coli*, as a negative control. 3–6: Cell lysates from transformed *E*. *coli* with recombinant P^Gex–4t1-VP2^, collected at different time points post IPTG induction (3 is the last, 6 is the initial); the VP2 apparent band revealed in different intensity over the time (boxed). (**B**) SDS-page visualizing the purified VP2 recombinant protein. 1: Protein marker, 2: cell lysate from non-transformed *E*. *coli*, as a negative control, 3: cell lysate from *E*. *coli* transformed with non-recombinant P^Gex–4t1^, as a positive control (express GST-protein at 26 kDa) (arrow), 4: cell lysate from *E*. *coli* transformed with recombinant P^Gex–4t1-VP2^ revealing the VP2 + GST (arrow) and 5: purified VP2-capsid protein after *thrombin* cleaving (22 KDa).

The recombinant plasmid harboring the VP2-coding sequence fused with GST-tag was transformed into *E*. *coli* BL21 cells. The recombinant VP2 was detected only in the lysates of bacteria transformed with the recombinant plasmid and was absent in lysates of the cells transformed with the empty P^Gex–4t1^ vector. The cells were harvested and analyzed at 1, 2, 3, and 16 h post IPTG induction. A putative VP2 band was identified as the expected molecular weight of about 48 kDa (22 KDa VP2-capsid + 26 KDa Gst-tag proteins) in SDS-page (Fig. 4A). The apparent GST-tagged VP2 band was positively detected by probing with the rabbit anti-GST antibody (not shown). The recombinant VP2 was purified by affinity-based chromatography using Sepharose under native conditions. After cleaving the GST-tag with *thrombin*, a band of the lower molecular weight of about 22 kDa was detected (Fig. 4B). The concentration of the purified VP2 was 4 mg of protein per liter of culture media.

### The recombinant VP2 as an antigen

#### Immunoreactivity and antigenicity

**Figure 5:**
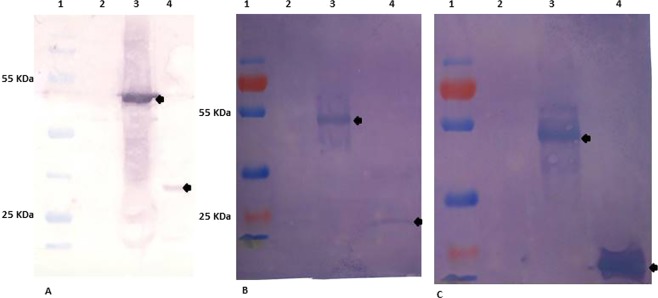
Western blot analysis of the VP2 immunoreactivity and antigenicity. VP2 immunoreactivity where it was able to detect FMDV antibodies in serum from trivalent vaccinated bovine (**A**), in addition to its ability to recognize specific anti-FMDV SAT 2 guinea pig antiserum (**B**), while its antigenicity was assessed by raising effective polyclonal antibodies in mice (**C**). 1: Protein marker, 2: cell lysate from non-transformed *E*. *coli* as a negative control, 3: cell lysate from *E*. *coli* transformed with recombinant P^Gex–4t–1-VP2^ and 4: purified VP2-capsid protein after being liberated by *thrombin* cleaving.

The western blot assay was used to assess rVP2 immunoreactivity with the FMDV Ab, either in serum from FMDV trivalent vaccinated animals (Fig. 5A), and the FMDV SAT 2 guinea pig antiserum, which is routinely used as tracing/detecting Ab in LPBE, (Fig. 5B), where strong positive signals were observed when the mice sera were reacted to the rVP2 either tagged with GST protein or after being liberated with *thrombin*.

**Figure 6:**
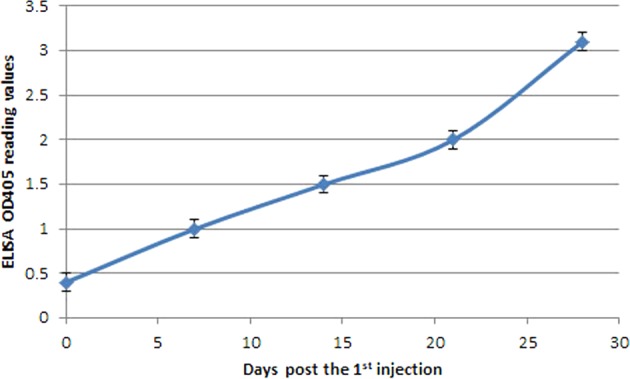
Indirect-ELISA showing the elevated immune response against VP2 in immunized mice serum. A significant increase in binding activity to the given antigen (rVP2) with sera samples taken at 0, 7, 14, 21, and 28 days (square points) post initial immunization. The error bar represents the value of 0.1 more or less of the ELISA reading value.

Furthermore, antigenicity of rVP2 was confirmed by its ability to raise anti-FMDV VP2 Ab in mice serum, where the specific seroconversion of mice was verified by western blot against either the purified rVP2 or bacterial cell lysate expressing the rVP2. The positive reactivity as represented by clear bands of the proper sizes was observed when the mice sera were reacted to the rVP2 either tagged with GST protein or after being liberated with *thrombin*. However, there were no detectable signals with lysates of *E*. *coli* cells transformed with an empty P^Gex–4t1^ vector (Fig. 5C). Furthermore, the indirect VP2-ELISA detected reasonable seroconversion in immunized mice sera with a significant sequential increase in the reactivity to the coating antigen (rVP2) with the sera collected on 0, 7, 14, 21, and 28 days post initial immunization (Fig. 6).

### Serotype-independent detection of VP2 protein

**Figure 7:**
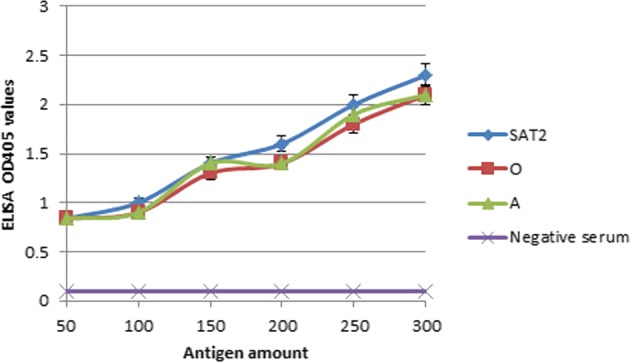
Indirect-ELISA showing the sensitivity of VP2 in capturing the antibodies against different FMDV serotypes (O, A, and SAT 2). Different amount of VP2 protein ranging from 50 to 300 ng was coated.

In order to determine if VP2 was recognizable by FMDV specific antibodies through ELISA, different amounts of VP2 were used as coating antigen in indirect ELISA to capture FMDV specific antibodies in sera from FMDV-infected animals representing the three serotypes O, A, and SAT 2. The results (Fig. 7) showed that the three serotypes reacted positively and gave high OD values compared to the negative control. A reasonably elevated signal with increasing VP2 concentration was noticed plus a non-significant variation among the three serotypes was detected.

### The sensitivity of VP2-based ELISA comparing with VNT and LPBE

Fifteen (*n* = 15) sera from naïve calves infected with respective serotypes O, A, and SAT 2 (*n* = 5 from each serotype). In addition, fourty five samples (*n* = 45) from naïve calves vaccinated with the local inactivated polyvalent vaccine, that were confirmed as positive by LPBE, exhibited virus neutralizing antibody titers ranging from log_10_ 1.37 to log_10_ 2.1 by VNT. When VNT data were correlated to their respective indirect VP2 ELISA OD values, there was no significant difference between the tests (R ≥ 0.98) (Table 1). Surprisingly, one naïve calf serum sample showed a positive reaction (doubtful) to VP2 with OD of 0.43 and 0.35 in one of the duplicate wells, when tested by the VP2 based ELISA. Therefore, the indirect ELISA recognized 23 of 24 true negative samples as negative, resulting in the specificity of 98.3% (Table 2). Regarding the true positive samples, the ELISA scored all 60 samples as positive, with a sensitivity of 100%.

**Table 1:** Summary of comparative mean reactivity of bovine sera tested by VNT, LPBE, and indirect VP2-ELISA.

Bovine Serum Samples	VP2-based ELISA (OD value)^*^	LPBE (Log_10_)^Ѱ^	VNT (Log_10_)^Ӌ^
**I**. **Positive Sera**			
A. Infected calf sera (n = 15)			
1- FMDV“O” infected	1.68 ± 0.43	1.77 ± 0.14	2.10 ± 0.12
2- FMDV“A” infected	1.59 ± 0.25	1.78 ± 0.09	1.66 ± 0.08
3- FMDV“SAT 2” infected	1.41 ± 0.17	1.53 ± 0.19	1.59 ± 0.26
B- vaccinated calf sera (n = 45)	1.77 ± 0.11	1.69 ± 0.09	1.87 ± 0.12
**II. Negative Sera**			
1- FCS (PAA, Austria), (n = 8)	0.13 ± 0.09	0.11 ± 0.05	0.15 ± 0.04
2- NCS (Sigma, Germany), (n = 8)	0.15 ± 0.09	0.21 ± 0.11	0.18 ± 0.09
3- NCS (HyClone, USA), (n = 8)	0.17 ± 0.05	0.19 ± 0.07	0.19 ± 0.07
4- Naïve healthy calves, (n = 37)	0.22 ± 0.07	0.21 ± 0.09	0.19 ± 0.07

The titers of sera from vaccinated calves and negative controls are the mean titers recorded against the three FMDV serotypes (A, O, and SAT 2) using VNT and LPBE.

*An OD value of ≥ 0.4 determined by the indirect rVP2-based ELISA was regarded as a positive sample.

^Ѱ^The Ab titers were expressed as the Log_10_ of the reciprocal of the highest dilution of the test serum giving ≤ 50% reactivity against rVP2.

^Ӌ^The positive anti-FMDV antibody titer ≥ 0.9 Log_10_ TCID_50_.

**Table 2:** Sensitivity and specificity of the indirect VP2-protein-based ELISA compared with the VNT.

	Indirect VP2-protein-based ELISA	VNT	LPBE
Sensitivity^a^	60/60 (100%)	60/60 (100%)	60/60 (100%)
Specificity^b^	60/61 (98.3%)	61/61 (100%)	61/61 (100%)

^a^It was assessed as the true positives versus the total number of positive serum samples.

^b^It was assessed as the true negatives versus the total number of negative serum samples.

### Standardization and application of VP2 protein-based ELISA

**Figure 8:**
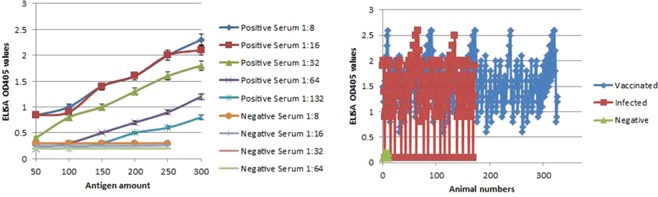
(**A**) Standardization of rVP2 protein-based ELISA by checkerboard titration between rVP2 protein and serum antibodies from naïve calves vaccinated with the local inactivated polyvalent O, A, and SAT 2 oil vaccine, in ELISA. Twofold serum dilution starting with a dilution of 1:8, and serial dilution of rVP2 ranged from 300 to 50 ng/well. The negative control reaction is PBS. (**B**) Applicability of rVP2-based ELISA showing the detection of FMDV antibodies in sera of clinical cases collected during field outbreaks (155 out of 172), and in naïve calves vaccinated with the local inactivated polyvalent O, A, and SAT 2 oil vaccine (328 out of 328), while FMDV free commercial sera used as controls did not result in any detectable signals.

In order to standardize the use of VP2-protein in indirect ELISA, the optimal antigen dilution was determined by using a checkerboard titration in which serial dilutions of antigen started with 300 ng/µL and were titrated against twofold dilutions of four positive sera (medians) from naïve calves vaccinated with the local inactivated polyvalent O, A, and SAT 2 vaccine, in addition to four negative sera from healthy animal (medians). The optimal VP2 dilution was chosen by considering the OD405 in the indirect ELISA, which demonstrated a maximum positive-to-negative ratio between positive and negative sera (Fig. 8A). The OD values were obtained with the 150 ng/well of VP2 protein and 1:32 of positive sera from vaccinated animals.

Furthermore, to check the applicability of the developed assay, the sera samples obtained from clinical cases of FMD field outbreaks (*n* = 172) and from the naïve calves vaccinated with the local inactivated polyvalent O, A, and SAT 2 oil vaccine (*n* = 328) were subjected to the detection by the recombinant VP2-based ELISA. All sera from vaccinated animals positively reacted with rVP2, while 155 of the 172 sera obtained from field clinical cases of FMD showed a positive reaction. The FMDV-free commercial control sera and fourteen sera from healthy animals did not result in any detectable signal, helping in determining the cut-off value (Fig. 8B).

## Discussion

FMDV serotypes differ considerably at their antigenic domains; this makes FMDV serodiagnosis complicated. Therefore, there is a decisive need for a reliable tool capable of monitoring seroconversion to FMDV regardless of its serotype. The aim of this study was to construct and express a recombinant structural protein (SP), i.e., VP2, which is a considerably conserved component of FMDV capsid, in a prokaryotic expression system to be utilized as a coating antigen in a simple indirect ELISA for detection of anti-FMDV antibodies (Ab) regardless of its serotype. In this study, an assay is developed for detection of antibodies against FMDV-SP in animal sera whether due to infection or vaccination. It is expected that this assay would be applicable for confirming previous or ongoing infection in non-vaccinated animals, to certify seronegative animals prior to transportation (trade purposes), and sero-monitoring of FMDV vaccinated animals. In Egypt, the open geographical boundaries, importing animals from regions with a history of FMDV diverse serotypes and the uncontrolled random backyard breeding represent a cumbersome facing FMD control programs. Consequently, animals that are not vaccinated on time remain as susceptible as foci of FMDV infection, that make it hard to follow up FMDV vaccination in such animals and to differentiate infected from vaccinated animals based on seroconversion. Recently, the Egyptian government has initiated a national wide animal numbering campaign in 2018, associated with FMDV vaccination to control this situation.

The currently used methods, viz., LPBE and VNT, have some drawbacks such as the use of potentially dangerous inactivated live virus, laborious, time-consuming, technically challenging, and have a significant rate of false positive results^15^. Although our developed assay is unable to distinguish diseased from vaccinated animals, it provides a serotype-independent detection of antibodies against FMDV in an ELISA format under laboratory conditions, where VNT cannot be applied as cell culture facility is not required. Therefore, the VP2-based ELISA, as a low-cost rapid pen-side test, could be utilized in a combination with other existing method like VNT and LBPE to rapidly assist diagnosis of an ongoing FMDV infection regardless its serotype.

Previous efforts on the development of an FMDV serotype-independent detection assay reported the use of an ELISA based on the seven serotype-based polyclonal antisera for FMDV diagnosis^16,17^. Further, a serotype-specific mAb-based ELISA^18^ and a competitive ELISA using mAb specific for the 12S subunit protein from six of the seven serotypes were developed for the detection of FMDV^19^. DAS-ELISA based on, two mAbs with different specificities^20^ and an assay based on mAbs produced through phage-displayed screening^21,22^ were proposed for type-independent FMDV diagnosis. However, the concept in all of the aforementioned attempts was based on the antigen detection, whereas our study is the first report of developing a serotype-independent detection method for FMDV Ab in given serum samples that could be from vaccinated or infected animals.

It has been established that the majority of the neutralizing epitopes of FMDV are localized on the VP1 capsid protein^13^. Thus it exhibits variable codons leading to VP1-based FMDV heterogeneity and emergence of new viral phenotypes^23–26^. Therefore, VP1 was excluded in our approach of developing a serotype-independent FMDV detection antigen. Interestingly, immunogenic epitopes on the B-C loop of VP2 protein have been reported in the previous studies^27^. Some studies suggested that the N terminal region of VP2, although exposed on the viral surface, has a low antigenic potential^28,29^. However, the N terminus of VP2 has been proven antigenic as it has no fixed position^14^, and its structural flexibility exposes some of the internal domains of the capsid proteins to the surface enabling them to become definite antigenic sites^30^. In a similar vein, another report^14^ showed that amino acid substitutions in the VP2 subunit result in a significant antigenic diversity that leads to changes in the pathogenicity and replication ability of FMDV. Thus, even if some sites of the VP2 N-termini are not accessible by Ab, the remaining accessible sites appear to be adequate for the detection by ELISA^30^. In addition to the antigenic properties of VP2, it has been confirmed that the N-terminal of VP2 is highly conserved among the FMDV serotypes^13^. Moreover, amino acid sequence alignments of the VP2 coding sequence revealed significant homology within FMDV serotypes, which was noticeable throughout the entire ORF, not only in the N-terminus region (data not shown). The reason behind selecting the entire ORF of VP2 for expression, rather than the N-terminus region, was to ensure proper folding of rVP2, and as such to preserve its antigenic determinants. We believe that selecting only the highly conserved N- terminus region of VP2, which has approximately 26 amino acid residues, could significantly affect protein folding and ultimately lead to protein aggregation. In our previous attempts, while trying to refold short peptides expressed in *E*. *coli*, we faced many challenges such as protein instability and aggregation. Others^31^ have also reported that the production and use of short peptides and proteins for pharmaceutical use are hampered by the formation of aggregates and protein deposition in *E*. *coli*. These data support our decision on the selection of VP2 protein to consider its antigenicity and specificity as an optional tool for FMDV serodiagnosis. In order to achieve this goal, we used the amino acid sequence of VP2 of the Egyptian local FMDV SAT 2 (gb│AAZ83686) as a model and aligned it with that of several FMDV isolates representing the seven prevalent FMDV subtypes to determine their relatedness. The sequence alignments revealed that VP2 capsid protein is highly conserved across the different FMDV subtypes. The VP2-coding sequence was then codon-optimized for its expression in *E*. *coli* and cloned in P^Gex–4t1^ as a fusion polypeptide with a GST tag. The VP2-GST-polypeptide (~48 kDa) was successfully expressed in *E*. *coli* and purified near homogeneity. After the liberation of VP2 protein from the GST, its immunoreactivity was confirmed by the western blot analysis against the FMDV antibodies found in the serum of FMDV trivalent vaccinated bovine and the guinea pig antiserum of FMDV SAT 2, which are routinely used as tracing/detecting antibodies in LPBE. Furthermore, western blot and indirect-ELISA evaluated the antigenicity of VP2 by confirming its ability for raising antibodies in mice serum. Additionally, using rVP2 protein as a coating antigen in indirect ELISA was able to capture FMDV specific antibodies in sera from FMDV-infected animals representing the three serotypes O, A, and SAT 2. Furthermore, the rVP2 based ELISA showed great sensitivity and specificity in detecting the FMD-specific antisera and the ELISA results were consistent with the VNT and LPBE with a high correlation (R ≥ 0.98). All FMDV positive sera from the infected and vaccinated calves had a mean virus neutralizing antibody (VN-Ab) titer of log_10_ ≥ 0.9 and mean OD value ≥ 0.4. The VNT test is considered as the “gold-standard” for FDMV diagnosis and also used as a quality control measurement for evaluating ELISA. The titer and mean OD value of anti-serotype O antibody were log_10_ 2.1 and ≥1.63, respectively, and were significantly higher (P > 0.05) than those obtained for the serotypes A and SAT 2 antisera. Similarly, the titer and mean OD value of anti-FMDV antisera collected from infected calves were significantly higher (P > 0.05) than those obtained from vaccinated calves. This discrepancy is likely due to the differences in the antigenicity of the different FMDV serotypes or due to the fact that the live FMDV induces faster and long-lasting immune responses than the attenuated FMDV. In order to standardize the use of VP2-protein in the indirect ELISA, the optimal antigen dilution was determined by using a checkerboard titration method. The optimal OD values were obtained with 150 ng/well of VP2 protein and 1:32 dilution of the positive sera from vaccinated animals. Furthermore, to check the applicability of the developed assay, the serum samples obtained from the clinical cases of FMD field outbreaks (*n* = 172) and from naïve calves vaccinated with the local inactivated polyvalent O, A, and SAT 2 oil vaccine (*n* = 328) were subjected to the detection by the rVP2-based ELISA. All sera from the vaccinated animals positively reacted with VP2, while 155 of the172 serum samples obtained from the clinical cases of FMD field outbreaks, showed a positive reaction.

The previous attempts to express FMDV capsids in *E*. *coli* always required a tedious re-folding procedure or resulted in poor solubility of the recombinant proteins^32^. In addition, the commercially available VP1 peptide-based ELISA from United Biomedical (NY, USA) is serotype specific, besides it does not detect antibodies against the structural proteins of the FMDV serotype O in the sera from animals vaccinated with a commercial vaccine^33^. In order to obtain optimal quality FMDV vaccines, suitable virus strains should be chosen on the basis of serological matching. The reactivity of the sera from vaccinated calves against field isolates should be measured by VNT or LPBE^34^. However, both, VNT and LPBE, are serotype specific and a positive result will be obtained only if the field and test strains have the same serotype. Furthermore, VNT requires special bio-containment for tissue culture system and biosafety precautions, since it is carried out with live FMDV. Therefore, the indirect VP2-based ELISA could offer an alternative, to some extent, or a companion pen-side test to the VNT and LPBE. Utility of the developed VP2-based ELISA at large was partly optimized as one strain per only three FMDV serotypes was studied. In fact, we studied the local isolates/strains available in Egypt as firm legislations prohibit introduction of any overseas strains. Also, it was really impossible to take samples from the same vaccinated animals to follow up the seroconversion in a given animal. That might be resolved by a follow up work in cooperation with an international FMD laboratory.

In summary, our study generated as the first time, a non-infectious antigen for the serotype-independent detection of FMDV antibodies using ELISA. The VP2-based ELISA has a great potential for adaptation to a rapid pen-side test, complementary to VNT and/or LPBE that can be carried out on-farm or at animal quarantines. This would reduce the use of inactivated FMDV antigen in diagnostic assays, which will also decrease the biosecurity risks and extend geographical acceptability of reagents for the assay. The recombinant VP2 capsid protein, which is a considerably conserved structural protein across FMDV serotypes, is found suitable ELISA coating antigen for serotype-independent rapid screening of sera from both infected and vaccinated animals. That might be of great value in quality control evaluation of polyvalent inactivated vaccines by rapid checking of animals prior to and after vaccination. Furthermore, its use will significantly lessen the time and expenses encountered for detection of seroconversion against different FMDV serotypes as well as to avoid the risky use of live viruses. Moreover, it highlights the utility of FMDV VP2-based ELISA for detecting FMDV Ab in sera from experimentally infected and vaccinated animals regardless the FMDV serotype, which would be helpful in confirming previous or ongoing infection in a non-vaccinated herd or population and to certify animals prior to movement for trade purposes.

## Methods

### Viruses, cells, and cultures

Egyptian local isolates of FMDV serotypes O (Manisa), A (Egy/2006), and SAT 2 (Egy/2012) were propagated in an appropriate bio-containment level at the quality control laboratory (QCL), Veterinary Sera and Vaccines Research Institute (VSVRI), Abbassia, Cairo, Egypt. BHK_21_ clone 13 cells from baby hamster kidney were used and maintained in minimum essential medium with Eagle’s salts (MEME) supplemented with heat-inactivated 5–10% newborn calf sera (NCS), 100 U/mL penicillin, 100 µg/mL streptomycin, and 25 i.u./mL mycostatin.

### Serum samples and antibodies

#### Negative sera

Newborn calf sera (HyClone, Northumberland, USA and Sigma, Germany) and fetal calf serum (PAA Laboratories, Pasching, Austria), in addition to one hundred sera, were collected from healthy animals. Before use, all negative sera were assured to be free of anti-FMDV antibodies by virus neutralization test^35^. The negative sera were utilized as controls to test the specificity of the developed VP2-ELISA antigen. Each negative control serum was tested eight times in quadruplicates to aid verification of the cut off value and to test the reproducibility.

#### Positive sera

A total of sixty positive sera (*n* = 60) were tested as experimental positive control sera to determine specificity and sensitivity of the developed assay: Fifteen sera (*n* = 15) obtained from naïve calves infected with serotypes O, A, and SAT 2, (*n* = 5 of each serotype), and forty-five sera (*n* = 45) from naïve calves vaccinated with the local inactivated trivalent O, A, and SAT 2 vaccine.

The selected sera were collected at different times after infection or vaccination representing a variety of anti-FMDV antibody titers.

#### Field sera

Three hundred twenty-eight (*n* = 328) serum samples were collected at four weeks post-vaccination from animals routinely vaccinated every four to six months with the trivalent inactivated vaccine from different governorates in Egypt. Besides, one-hundred seventy-two (*n* = 172) field sera from clinical cases of FMDV that were collected at different clinical stages during some outbreaks from 2006 to 2016 were also tested. These field sera were included in order to evaluate reproducibility and applicability of the developed VP2-based ELISA.

#### Commercial antibodies

Guinea-pig antiserum of O Manisa (DDT-FMDV-O-GP), A22 (DDT-FMDV-A-GP), and SAT 2 (DDT-FMDV-SAT2-GP) from Pirbright were used. Anti-Bovine-HRP, (A18751) and Anti-Guinea Pig-HRP (PA1–28597) from ThermoScientific in addition to Anti-Bovine-ALP (A0705) and Anti-Guinea-ALP, (A5062) from Sigma-Aldrich were also used.

#### Sequence design

The sequence of FMDV Egyptian SAT 2 isolate (gb│AAZ83686) was used as the model and that of several isolates of different FMDV serotypes were retrieved from GenBank database and used in homology alignments to identify the highly conserved regions. Following multiple sequence alignments, a phylogenic tree was created using the ClustalW algorithm (version 5.0). The Jpred4 database was used to predict the secondary structure of the VP2-capsid protein (residues, 1–160) of the Egyptian SAT 2 isolate (gb│AAZ83686). The coding sequence of this protein was modified and codon optimized for expression in *E*. *coli*. The modified sequence was synthesized by GenScript, Piscataway, NJ, USA.

#### Gene expression

The VP2-coding sequence was amplified by PCR and sub-cloned in P^Gex–4t1^ (Invitrogen). Two specific primers, sense: 5′- *GGATCC*ACCACCACAAGCACCACC-3′, and the anti-sense: 5′-*CTCGAG*ATTACG GCAGTTCACCC-3′) flanked by *Bam*HI and *Xho*I sequences (*italic*), respectively, were designed. *E*. *coli* BL21 (DE3) competent cells prepared using the CaCl_2_ method^36^ were transformed with the recombinant P^Gex-4t1+VP2^ vector. Recombinant VP2 expression and purification were carried out according to the instructions provided in the Glutathione Sepharose 4B GST-tagged protein purification resin kit, GE Healthcare, life sciences. Briefly, an overnight culture of a clone harboring P^Gex–4t1+VP2^ vector was inoculated into fresh LB broth medium containing ampicillin, and grown at 37 °C until OD_600_ reached 0.6. The culture was then induced by IPTG (1 mM), and then re-incubated for an additional 3 h at 28 °C. The bacteria were harvested by centrifugation at 4000 × g for 20 min and re-suspended in lysis buffer (50 mM NaH_2_PO_4_, 300 mM NaCl, pH 8.0). The cells were lysed by subjecting to one freeze-thaw cycle (−80 °C/37 °C) and then sonicated on ice for a few seconds. Clear lysates containing the GST-VP2- fusion polypeptide were affinity purified under native conditions using Sepharose beads. The bound VP2-GST-fusion protein was eluted from the Sepharose beads, and the GST-tag was cleaved using *Thrombin* enzyme. VP2 expression and purity were assessed by 12% SDS-PAGE^37^, and its concentration was estimated by Bradford assay^38^.

#### Mice immunization

Eight-week-old female *Balb/C* mice obtained from Theodor Bilharz Research Institute, Egypt, were housed in pathogen-free conditions for six weeks and given access to food and water. The mice were treated in accordance with the principles and policies of the National Institute of Health (NIH) animal care. All experiments were approved by the research ethics committee, National Center for Radiation Research and Technology (REC-NCRRT/13 A/18). The mice were injected intraperitoneally with 30 μg of recombinant VP2 protein dissolved in 0.01 M Tris-HCl buffer (pH 8) emulsified in Freund’s complete adjuvant to elicit the primary response. This was followed by four weekly booster injections of 200 μg protein in Freund’s incomplete adjuvant. The mice were observed daily and euthanized humanely at the end of the immunization regime and their blood was collected.

### VP2 antigenicity

#### Indirect ELISA

VP2 antigenicity was determined by assessing the specific antibodies raised in the collected serum of the immunized mice by indirect ELISA. Briefly, a 96-well ELISA plate (Nunc, Denmark) was coated with 50 ng/well of rVP2 protein diluted in carbonate and bicarbonate buffer pH 9.6 at 4 °C/overnight. The wells were washed with Phosphate-Buffered Saline (PBS) containing 0.01% Tween, then bovine serum albumin (BSA) (3%) was used to block the free residual-spaces. The wells were washed twice with PBST; serum collected before each booster was diluted in PBS (1:3000) and added to the wells and incubated for 2 h at room temperature (RT). The zero-day mice sera (prior to the first mice-immunizations) were collected and used as negative controls for this assay. The wells were intensively-washed with PBST. The rabbit anti-mouse IgG (Sigma Aldrich) conjugated with alkaline phosphatase (ALP) was added at a dilution of 1:20000 and incubated for 2 h at RT. The excess and unbound conjugated antibodies were removed by washing with phosphate buffer saline-Tween (PBST) and the reaction was developed by 100 μL of p-Nitrophenyl Phosphate (PNPP)-substrate solution. To stop the reaction, 3 M NaOH was added and the absorbance was determined at 450 nm using Bio-Rad I Mark microplate reader.

#### Western blot

Furthermore, the VP2 antigenicity was also analyzed by western blot. Briefly, proteins were separated on 12% SDS-PAGE^37^ and blotted on a nitrocellulose membrane, followed by blocking with 4% BSA at 4 °C overnight. After washing with TBS containing 0.1% Tween, mouse raised antibodies diluted at 1:10000 in TBS were added and incubated for 2 h with gentle shaking at RT. After washing, goat anti-mouse antibodies conjugated with ALP (Sigma, USA), diluted at 1:20000, were added as secondary antibodies and incubated for 1 h at RT. The membrane was washed again as previously described and color reaction was developed with nitro-blue tetrazolium chloride (NBT) and 5-bromo-4-chloro-3′-indolyphosphate p-toluidine salt (BCIP) solution. Total cell lysate of *E*. *coli* BL21 transformed with non-recombinant P^Gex–4t1^ was used as a negative control.

### VP2-immunoreactivity against the antibodies of FMDV

#### Indirect ELISA

The sensitivity of VP2-protein to capture the FMDV antibodies in the serum of infected animals was assessed by indirect ELISA, which was basically performed as described above except for different concentrations of VP2 protein, ranging from 500 to 100 ng per well, were coated in ELISA plate. Three of the positive sera from naïve calves infected with FMDV O, A, and SAT 2 serotypes, respectively, were individually diluted in PBS containing 3% BSA, seeded at 100 µL per well in duplicate (4 times) and incubated for 2 h at 37 °C. After decanting the sera and washing with anti-bovine IgG (KPL, MD, USA) labeled with horseradish peroxidase diluted at 1:1000 in PBS containing 3% BSA was added (100 µL/well). After incubation at 37 °C for 1 h, the plates were decanted and washed, and 100 µL TMB-ELISA substrate (KPL, MD, USA) was added per well. A microplate reader (Vmax kinetic) was used to read the plates at 450 nm.

#### Western blot

VP2 immunoreactivity against FMDV antibodies in serum from trivalent vaccinated bovine in addition to the specific guinea pig anti-FMDV SAT 2, usually used as a tracing antibody, was analyzed using western blot exactly as previously described. Anti-Bovine-ALP was used for detection.

#### Liquid phase blocking ELISA

All control sera either negative or positive were verified for anti-FMDV structural protein antibodies by liquid phase blocking ELISA (LPBE) as described earlier^8,9^. Two-fold dilutions of each serum (from a dilution of 1:16 to 1:128) were tested for FMDV O, A, and SAT 2 serotype-specific antibodies. To determine the background reactivity, four wells were used without test sera as antigen controls for 0% inhibition, and two wells were used without any antigen as conjugate control. The Ab titers were expressed as the Log_10_ of the reciprocal of the highest dilution of the test serum giving ≤ 50% reactivity against rVP2.

#### Virus neutralization assay

Virus Neutralization test (VNT) was conducted in micro-titration plates as described earlier^35^. Briefly, two-fold dilutions of heat-inactivated sera (56 °C for 30 min) were incubated with 100 µL TCID50 at titers of 108.75, 108, and 107.5 of each FMDV A, O and SAT 2 serotype and 100 µL BHK21 cells. All samples were tested four times, each time in duplicate, and the final readings were recorded after two days of incubation at 37 °C. The titers of virus neutralizing antibody in every sample were calculated as log10 of the reciprocal of that dilution of serum that resulted in ≥ 50% cells with no cytopathic effect. The neutralizing antibody titer <1:4 (< 0.6 log10) was considered negative for a serum sample.

#### Standardization and application of VP2-protein based indirect ELISA

To standardize the use of VP2-protein-based ELISA and to determine the optimal antigen dilution, indirect ELISA was performed as the established protocol^26^ with minor modifications. Several checker-board titrations, serum dilutions, pH, buffers and reactant volumes and concentrations were tested for maximizing specificity and sensitivity and minimizing background. The optimal antigen dilution was determined by using a checkerboard titration in which twofold dilutions of antigen starting with 100 ng/well were titrated against twofold dilutions of four positive sera from naïve calves vaccinated with the local inactivated polyvalent O, A, and SAT 2 vaccine, in addition to four negative sera from healthy animals. The recombinant VP2-protein was diluted in PBS buffer plus 0.05% Triton X-100. ELISA plates (Nunc, IL, USA) were coated with 100 µL of the diluted VP2-protein (0.04 µg/well) per well and incubated at 4 °C overnight. The wells were washed with PBST (0.1% Tween 20), blocked at 37 °C for 2 h with 100 µL per well-blocking buffer (PBS, pH 7.4 plus non-fat milk 5% and bovine serum and BSA 3%). After removing the blocking buffer, the plates were washed as described. Each serum sample was diluted in PBS containing 3% BSA, seeded at 100 µL per well in duplicate (4 times) and incubated for 2 h at 37 °C. After decanting the sera and washing, anti-bovine IgG (KPL, MD, USA) labeled with horseradish peroxidase diluted 1:1000 in PBS containing 3% BSA was added (100 µL/well). After incubation at 37 °C for 1 h, the plates were decanted and washed, and 100 µL TMB-ELISA substrate (KPL, MD, USA) was added per well. A microplate reader (Vmax kinetic) was used to read the plates at 450 nm, and the absorbance value (OD) of 0.4 was determined as the cutoff point. The samples with OD ≥ 0.4 were considered positive, 0.3–0.4 as doubtful (should be repeated), and ≤ 0.3 as negative.

Furthermore, to verify the diagnostic value of VP2-protein-based ELISA, it was applied for detecting FMDV antibodies (*n* = 172) in the sera obtained from clinical cases of FMD field outbreaks and from naïve calves vaccinated with the local inactivated polyvalent O, A, and SAT 2 oil vaccine (*n* = 328).
